# Association of serum cortisol with insulin secretion and plasma aldosterone with insulin resistance in untreated type 2 diabetes: a cross-sectional study

**DOI:** 10.1186/s13098-025-01706-8

**Published:** 2025-04-28

**Authors:** Masahiro Ohira, Naoyuki Kawagoe, Chisato Kameyama, Yuko Kondou, Madoka Igarashi, Hajime Ueshiba

**Affiliations:** Division of Diabetes, Metabolism and Endocrinology, Department of Internal Medicine, Toho University Ohashi Medical Center, 2-22-36 Ohashi, Meguro-ku, Tokyo, 153-8515 Japan

**Keywords:** Cortisol, Aldosterone, Insulin secretion, Insulin resistance, Untreated type 2 diabetes

## Abstract

**Background:**

Insulin secretion and resistance are key pathophysiological factors in type 2 diabetes. However, only 55% of patients achieve long-term blood glucose treatment goals, highlighting the need to clarify the pathophysiology of type 2 diabetes. While cortisol and aldosterone levels have been linked to insulin secretion and resistance in participants without type 2 diabetes, their role in patients with type 2 diabetes remains unclear. In this study, we aimed to investigate the relationships among insulin secretion, insulin resistance, and cortisol or aldosterone levels in patients with untreated type 2 diabetes.

**Methods:**

We retrospectively reviewed 121 patients with untreated type 2 diabetes mellitus. We analyzed the relationships between various clinical parameters, including adrenal hormones, and insulin secretion (homeostatic model assessment [HOMA2-%B]) or insulin resistance (HOMA2-IR). Multiple regression analysis was performed to identify parameters associated with HOMA2-%B or HOMA2-IR.

**Results:**

Spearman’s rank correlation coefficient revealed that body weight (BW); body mass index (BMI); estimated glomerular filtration rate; and serum creatinine, uric acid, total cholesterol, high-density lipoprotein cholesterol (HDL-C), sodium, potassium, chloride, fasting blood glucose (FBG), glycated hemoglobin (HbA1c), serum C-peptide, and cortisol levels were significantly correlated with HOMA2-%B. Similarly, BW, BMI, aspartate transaminase levels, alanine transaminase (ALT) levels, triglyceride levels, HDL-C levels, FBG levels, serum C-peptide levels, renin activity, and plasma aldosterone concentration (PAC) were significantly correlated with HOMA2-IR. Multiple regression analysis revealed BMI, HbA1c levels, and cortisol levels as predictors of HOMA2-%B, whereas ALT levels and the PAC were predictors of HOMA2-IR.

**Conclusion:**

Serum cortisol levels are associated with insulin secretion, and the PAC is associated with insulin resistance in patients with untreated type 2 diabetes. These findings suggest that aldosterone blockade may represent a potential therapeutic approach for reducing insulin resistance in patients with type 2 diabetes.

## Background

Insulin secretion and resistance are key pathophysiological factors in type 2 diabetes [1]. Both glucotoxicity and lipotoxicity are known to impair insulin secretion [2]. Furthermore, other factors, such as aging and tumor necrosis factor (TNF)-alpha (α), contribute to impaired insulin secretion [2]. In contrast, obesity is a major factor that exacerbates insulin resistance [3]. TNF-α, resistin, interleukin-6, and retinol-binding protein 4 inhibit insulin sensitivity [4]. Some factors related to insulin secretion and resistance have been identified; however, only 55% of patients with type 2 diabetes achieve long-term blood glucose treatment goals [5, 6]. Therefore, managing type 2 diabetes remains challenging.

Cortisol and aldosterone also play important roles in hyperglycemia. For example, cortisol is a significant hyperglycemic factor [7], and patients with primary aldosteronism have a higher incidence of diabetes [8]. Higher serum cortisol levels are associated with elevated fasting plasma glucose levels and reduced β-cell function in African Americans without type 2 diabetes [9]. Similarly, higher serum cortisol levels are associated with decreased insulin secretion in the general Japanese population [10]. Higher aldosterone levels are associated with increased insulin resistance and incidence of type 2 diabetes in individuals without type 2 diabetes [11]. Furthermore, plasma aldosterone levels are correlated with hyperinsulinemia incidence and insulin resistance in patients with hypertension without diabetes [12]. Thus, cortisol and aldosterone levels are associated with insulin secretion and/or insulin resistance in individuals without type 2 diabetes. However, the relationships between cortisol or aldosterone levels and insulin secretion and/or resistance in patients with untreated type 2 diabetes remain unclear. If cortisol and/or aldosterone levels are related to insulin secretion and/or resistance in patients with type 2 diabetes, these two hormones might represent novel therapeutic targets. Therefore, in this study, we aimed to investigate the relationships between insulin secretion and/or insulin resistance and between cortisol or aldosterone levels in patients with untreated type 2 diabetes.

## Methods

### Study design and participants

We retrospectively reviewed clinical data obtained from March 2021 to February 2023 at Toho University Ohashi Medical Center (Meguro-ku, Tokyo, Japan) to identify patients with untreated type 2 diabetes. This study aimed to clarify the relationships between adrenal hormones and insulin secretion and/or resistance. Pharmacotherapies for type 2 diabetes influence insulin secretion of/and resistance. Therefore, we selected patients with untreated type 2 diabetes for this study. Patients were diagnosed with type 2 diabetes if their glycated hemoglobin (HbA1c) and fasting blood glucose (FBG) levels exceeded 6.5% and 126 mg/dL, respectively, and if they did not test positive for specific antibodies associated with type 1 diabetes. We excluded patients who met the following criteria: did not have cortisol or aldosterone level measurement data(*n* = 18); were already receiving glucocorticoids for the treatment of other diseases (*n* = 6); were diagnosed with diabetic ketosis or ketoacidosis (*n* = 4); were taking angiotensin-converting enzyme inhibitors, angiotensin receptor blockers, β-blockers, or mineralocorticoid receptor antagonists (*n* = 10); and had blood glucose levels exceeded 450.5 mg/dl (*n* = 6) because the homeostatic model assessment (HOMA)2 cannot calculate steady-state β-cell function (HOMA2-%B) and HOMA2-insulin resistance (IR) when blood glucose levels are above this threshold (fasting blood glucose concentration across a range of 1–25 mmol/L) [13].

During the study period, 165 patients with untreated type 2 diabetes visited our hospital. After applying the exclusion criteria, 44 patients were excluded, and 73.3% (*n* = 121) of the patients were included in the study.

The following parameters were measured: body weight (BW), body mass index (BMI), aspartate transaminase (AST) levels, alanine transaminase (ALT) levels, blood urea nitrogen (BUN) levels, serum creatinine levels, estimated glomerular filtration rate (eGFR), uric acid (UA) levels, total cholesterol (TC) levels, triglyceride (TG) levels, high-density lipoprotein-cholesterol (HDL-C) levels, low-density lipoprotein cholesterol levels, FBG levels, HbA1c levels, serum C-peptide levels, HOMA2-%B, HOMA2-IR, and blood pressure (BP). To identify secondary diabetes, the levels of plasma adrenocorticotropic hormone (ACTH) and serum cortisol, plasma renin activity, and the plasma aldosterone concentration (PAC) were measured. At our hospital, BW measurements and blood sample collection are typically performed in a fasting state, and blood samples for measuring plasma renin and aldosterone are collected after the patient has rested for 30 min in a supine position.

### Measurements of various parameters

Within 1 h of blood collection, the serum and plasma were separated by centrifuging the samples at 3000 rpm for 10 min. The serum and plasma levels were used to measure the HbA1c, AST, ALT, BUN, creatinine, eGFR, lipid, C-peptide, and hormone levels. Plasma ACTH levels were measured via an electrochemiluminescence immunoassay (ECLIA) using an ECLusys^®^ reagent ACTH assay kit (Roche Diagnostics, Basel, Switzerland).

Serum cortisol levels were measured via a chemiluminescence immunoassay (CLIA) using a Cortisol Abbott assay (Abbott, Chicago, IL, USA). ECLIA was performed using the cobas8000 system (Roche Diagnostics, Basel, Switzerland). CLIA was performed using the Architect i2000 SR system (Abbott, Chicago, IL, USA). Plasma renin activity was measured via enzyme immunoassay (EIA) using a YAMASA^®^ Renin Activity Kit (YAMASA CORPORATION, Chiba, Japan). EIA was performed using the AP-X system (Hitachi Chemical Company Ltd., Tokyo, Japan). The PAC was measured via ECLIA using a Lumipulse^®^ Presto aldosterone assay kit (Fujirebio, Tokyo, Japan). All measurements of ACTH, cortisol, aldosterone levels and renin activity were conducted at LSI Medience Corporation (Tokyo, Japan).

### Calculation of HOMA2-%B and HOMA2-IR

We used HOMA2, an updated HOMA model, to evaluate insulin secretion and resistance in this study. HOMA2 accounts for variations in hepatic and peripheral glucose resistance [13]. HOMA2-%B and HOMA2-IR were calculated using the HOMA2 Calculator (Oxford Centre for Diabetes, Endocrinology and Metabolism, Oxford, United Kingdom). The calculations were based on FBG (mg/dL) and serum C-peptide (ng/mL) levels.

### Statistical analysis

The normality of the data distribution was tested using the Shapiro–Wilk test. Continuous data are expressed as medians and interquartile ranges (IQRs), as many datasets were nonnormally distributed. The correlations between HOMA2-%B or HOMA2-IR and each clinical parameter were assessed using Spearman’s rank correlation coefficient. Multiple regression analysis was performed to analyze the independent associations of the variables with HOMA2-%B or HOMA2-IR. Clinical factors that correlated significantly with HOMA2-%B or HOMA2-IR according to Spearman’s rank correlation analysis were selected for the regression models. FBG and serum C-peptide levels were excluded from both models because HOMA2-%B and HOMA2-IR are calculated using these parameters. Multicollinearity was assessed using the variance inflation factor (VIF). For HOMA2-%B, the following parameters showed significant correlations: BW; BMI; eGFR; and the levels of serum creatinine, UA, TC, HDL-C, sodium, potassium, chloride, FBG, HbA1c, serum C-peptide, and cortisol. BW and serum creatinine levels were excluded from the model because the VIF was **>** 5. Therefore, the final model for HOMA2-%B included BMI; eGFR; and the levels of UA, TC, HDL-C, sodium, potassium, chloride, HbA1c, and cortisol. For HOMA2-IR, the following parameters were significantly correlated: BW; BMI; the levels of AST, ALT, TG, HDL-C, FBG, and serum C-peptide; renin activity; and the PAC. BW and AST levels were excluded from the model because the VIF was **>** 5. Therefore, the final model for HOMA2-IR included BMI, ALT levels, TG levels, HDL-C levels, renin activity, and the PAC. Statistical significance was set at *P* < 0.05. All the statistical analyses were performed using JMP Pro software (version 17.2.0; SAS, Cary, NC, USA).

## Results

### Participant characteristics

Table 1 presents the characteristics of the participants. The median (IQR) age, BMI, and HbA1c level were 51.0 (44.0–64.0) years, 25.4 (22.9–28.8) kg/m^2^, and 10.3 (7.9–12.3)%, respectively. The median (IQR) values for HOMA2-%B and HOMA2-IR were 32.6 (21.3–64.7) and 2.3 (1.5–3.3), respectively. Systolic and diastolic BP were within normal ranges {systolic BP: 134.0 (122.0–146.5), diastolic BP: 80 (68.5–89.5)}. The median (IQR) values for ACTH and cortisol levels were 26.0 (16.4–41.0) pg/mL and 13.1 (9.5–16.6) µg/mL, respectively. The plasma renin activity and PAC were 1.6 (0.6–3.2) ng/mL/h and 59.9 (32.2–102.0) pg/mL, respectively. The aldosterone/renin activity ratio (ARR) was 36.8 (18.3–81.1), which was below the threshold of 200 (Table 1). The remaining parameters are listed in Table 1.

**Table 1:** Participant characteristics (*N* = 121)

Sex (male/female) Age (years) Family history of type 2 diabetes (%) BW (kg) BMI (kg/m^2^) AST (IU/L) ALT (IU/L) BUN (mg/dL) Serum creatinine (mg/dL) eGFR (mL/min/1.73m^2^) Uric acid (mg/dL) TC (mg/dL) TG (mg/dL) HDL-C (mg/dL) LDL-C (mg/dL) Sodium (mEq/L) Potassium (mEq/L) Chloride (mEq/L) FBG (mg/dL) HbA1c (%) Serum C-peptide (ng/mL) HOMA2-%B (%) HOMA2-IR Systolic BP (mmHg) Diastolic BP (mmHg) ACTH (pg/mL) Cortisol (µg/mL) Renin activity (ng/mL/h) Aldosterone (pg/mL) Aldosterone/renin activity	92 (76.0%)/29 (24.0%) 51.0 (44.0–64.0) 76 (62.8%) 72.0 (62.5–83.9) 25.4 (22.9–28.8) 22.0 (17.0–34.5) 25.0 (16.0–52.0) 13.0 (11.0–16.0) 0.73 (0.63–0.86) 82.5 (68.5–97.7) 5.4 (4.2–6.3) 207.0 (178.0–240.5) 149.0 (100.5–224.5) 49.0 (43.0–58.5) 126.0 (99.0–156.0) 140.0 (138.0–142.0) 4.0 (3.8–4.2) 102.0 (99.5–104.0) 197.0 (138.0–262.5) 10.3 (7.9–12.3) 2.3 (1.6–3.2) 32.6 (21.3–64.7) 2.3 (1.5–3.3) 134.0 (122.0–146.5) 80.0 (68.5–89.5) 26.0 (16.4–41.0) 13.1 (9.5–16.6) 1.6 (0.6–3.2) 59.9 (32.2–102.0) 36.8 (18.3–81.1)

Data are presented as the median and interquartile range (IQR). BW, body weight; BMI, body mass index; AST, aspartate transaminase; ALT, alanine transaminase; BUN, blood urea nitrogen; eGFR, estimated glomerular filtration rate; TC, total cholesterol; TG, triglycerides; HDL-C, high-density lipoprotein-cholesterol; LDL-C, low-density lipoprotein cholesterol; Eq, equivalent; FBG, fasting blood glucose; HbA1c, glycosylated hemoglobin; HOMA, homeostatic model assessment; BP, blood pressure; ACTH, adrenocorticotropic hormone

### Correlations between HOMA2-%B and each clinical parameter

Table 2 shows the correlations between HOMA2-%B and each of the clinical parameters. BW, BMI, serum creatinine levels, UA levels, sodium levels, chloride levels, and serum C-peptide levels were significantly positively correlated with HOMA2-%B (Table 2). In contrast, eGFR, TC levels, HDL-C levels, potassium levels, FBG levels, HbA1c levels, and cortisol levels were significantly negatively correlated with HOMA2-%B (Table 2). In summary, BW, BMI, serum creatinine levels, eGFR, UA levels, TC levels, HDL-C levels, sodium levels, potassium levels, chloride levels, FBG levels, HbA1c levels, serum C-peptide levels, and cortisol levels were significantly correlated with HOMA2-%B (Table 2). The other variables were not significantly correlated.

**Table 2:** Correlation between HOMA2-%B and each preoperative clinical parameter

	HOMA2-%B	
	ρ	P value
Sex (male;0, female;1) Age (years) Family history of type 2 diabetes BW (kg) BMI (kg/m^2^) AST (IU/L) ALT (IU/L) BUN (mg/mL) Serum creatinine (mg/mL) eGFR (mL/min/1.73m^2^) UA (mg/dL) TC (mg/dL) TG (mg/dL) HDL-C (mg/dL) LDL-C (mg/dL) Sodium (mEq/L) Potassium (mEq/L) Chloride (mEq/L) FBG (mg/dL) HbA1c (%) Serum C-peptide (ng/mL) HOMA2-IR Systolic BP (mmHg) Diastolic BP (mmHg) ACTH (pg/mL) Cortisol (µg/mL) Renin activity (ng/mL/h) Aldosterone (pg/mL) Aldosterone/renin activity	-0.1510 0.1192 -0.1168 0.2460 0.2639 0.1688 0.1074 0.1688 0.3028 -0.2976 0.3559 -0.2307 -0.1644 -0.1886 -0.0735 0.4906 -0.2577 0.6013 -0.8004 -0.7756 0.5721 0.1397 -0.0872 -0.0966 0.0065 -0.3148 -0.0752 -0.0886 0.0259	0.0982 0.1928 0.2022 0.0065 0.0034 0.0642 0.2410 0.0643 0.0007 0.0009 < 0.0001 0.0109 0.0715 0.0383 0.4248 < 0.0001 0.0043 < 0.0001 < 0.0001 < 0.0001 < 0.0001 0.1265 0.3414 0.2917 0.9439 0.0004 0.4120 0.3340 0.7783

HOMA, homeostatic model assessment; BW, body mass index; BMI, body mass index; AST, aspartate transaminase; ALT, alanine transaminase; BUN, blood urea nitrogen; eGFR, estimated glomerular filtration rate; UA, uric acid; TC, total cholesterol; TG, triglycerides; HDL-C, high-density lipoprotein-cholesterol; LDL-C, low-density lipoprotein cholesterol; Eq, equivalent; FBG, fasting blood glucose; HbA1c, glycosylated hemoglobin; BP, blood pressure; ACTH, adrenocorticotropic hormone

### Correlations between HOMA2-IR and each clinical parameter

Table 3 shows the correlations between the HOMA2-IR score and each clinical parameter. BW, BMI, AST levels, ALT levels, TG levels, FBG levels, serum C-peptide levels, renin activity, and the PAC were significantly positively correlated with HOMA2-IR (Table 3). In contrast, HDL-C levels were significantly negatively correlated with HOMA2-IR (Table 3). In summary, BW, BMI, AST levels, ALT levels, TG levels, HDL-C levels, FBG levels, serum C-peptide levels, renin activity, and the PAC were significantly correlated with HOMA2-IR (Table 3). The other variables were not significantly correlated.

**Table 3:** Correlations between HOMA2-IR and each preoperative clinical parameter

	HOMA2-IR	
	ρ	P value
Sex (male;0, female;1) Age (years) Family history of type 2 diabetes BW (kg) BMI (kg/m^2^) AST (IU/L) ALT (IU/L) BUN (mg/mL) Serum creatinine (mg/mL) eGFR (mL/min/1.73m^2^) UA (mg/dL) TC (mg/dL) TG (mg/dL) HDL-C (mg/dL) LDL-C (mg/dL) Sodium (mEq/L) Potassium (mEq/L) Chloride (mEq/L) FBG (mg/dL) HbA1c (%) Serum C-peptide (ng/mL) HOMA2-%B (%) Systolic BP (mmHg) Diastolic BP (mmHg) ACTH (pg/mL) Cortisol (µg/mL) Renin activity (ng/mL/h) Aldosterone (pg/mL) Aldosterone/renin activity	-0.1405 -0.1332 0.1650 0.4099 0.4111 0.3671 0.4540 0.0093 0.1701 -0.1242 0.1362 0.0469 0.3770 -0.1919 0.0314 -0.0.958 0.0230 -0.1778 0.4065 0.0176 0.8527 0.1397 0.0981 0.1696 -0.0458 0.0059 0.2984 0.2504 -0.0652	0.1242 0.1452 0.0705 < 0.0001 < 0.0001 < 0.0001 < 0.0001 0.9194 0.0621 0.1747 0.1381 0.6094 < 0.0001 0.0350 0.7336 0.2958 0.8022 0.0510 < 0.0001 0.8482 < 0.0001 0.1265 0.2844 0.0629 0.6182 0.9491 0.0009 0.0056 0.4777

HOMA, homeostatic model assessment; BW, body mass index; BMI, body mass index; AST, aspartate transaminase; ALT, alanine transaminase; BUN, blood urea nitrogen; eGFR, estimated glomerular filtration rate; TC, total cholesterol; TG, triglycerides; HDL-C, high-density lipoprotein-cholesterol; LDL-C, low-density lipoprotein cholesterol; Eq, equivalent; FBG, fasting blood glucose; HbA1c, glycosylated hemoglobin; BP, blood pressure; ACTH, adrenocorticotropic hormone

### Associations between HOMA2-%B and dependent variables

Table 4 summarizes the results of a multiple regression analysis examining the associations between HOMA2-%B and other clinical variables. The HbA1c level was the major independent predictor of HOMA2-%B, with BMI and cortisol levels also identified as independent predictors (HbA1c: standardized β=-0.4872, *P* < 0.0001; BMI: standardized β = 0.2041, *P* = 0.0067; cortisol: standardized β=-0.1798, *P* = 0.0089). Other baseline variables were not independent predictors of HOMA2-%B (Table 4).

**Table 4:** Correlations of HOMA2-%B with other variables analyzed via multiple regression models

	Standardized β	SE	*P* value
BMI (kg/m^2^) eGFR (mL/min/1.73m^2^) UA (mg/dL) TC (mg/dl) HDL-C (mg/dl) Sodium (mEq/L) Potassium (mEq/L) Chloride (mEq/L) HbA1c (%) Cortisol (µg/mL)	0.2041 -0.1534 0.0412 -0.0006 -0.0581 -0.0184 0.0272 0.1568 -0.4872 -0.1798	0.6034 0.1582 2.1180 0.0572 0.2241 1.4242 9.2610 1.3451 1.2851 0.5648	0.0067 0.0516 0.5979 0.9933 0.4386 0.8744 0.7178 0.1705 < 0.0001 0.0089

Model: r^2^ = 0.5365, *p* < 0.0001. HOMA, homeostatic model assessment; SE, standard error; BMI, body mass index; eGFR, estimated glomerular filtration rate; UA, uric acid; TC, total cholesterol; HDL-C, high-density lipoprotein-cholesterol; HbA1c, glycosylated hemoglobin

### Associations between HOMA2-IR and dependent variables

Table 5 summarizes the results of a multiple regression analysis examining the associations between HOMA2-IR and other clinical variables. The ALT level and PAC were found to be independent predictors of HOMA2-IR (ALT: standardized β = 0.1920, *P* = 0.0366; PAC: standardized β = 0.1753, *P* = 0.0458). Other baseline variables were not independent predictors of HOMA2-IR (Table 5).

**Table 5:** Correlations of HOMA2-IR with other variables analyzed via multiple regression models

	Standardized β	SE	*P* value
BMI (kg/m^2^) ALT (IU/L) TG (mg/dL) HDL-C (mg/dL) Renin activity (ng/mL/h) Aldosterone (pg/mL)	0.0937 0.1920 -0.0151 0.0559 -0.0082 0.1753	0.2924 0.0421 0.0066 0.1098 0.2381 0.0229	0.3624 0.0366 0.8846 0.5955 0.9326 0.0458

Model: r^2^ = 0.0990, *p* = 0.0479. HOMA, homeostatic model assessment; SE, standard error; BMI, body mass index; TG, triglyceride; HDL-C, high-density lipoprotein cholesterol

## Discussion

In the present study, BW; BMI; eGFR; and the levels of serum creatinine, UA, TC, HDL-C, sodium, potassium, chloride, FBG, HbA1c, serum C-peptide, and cortisol were significantly correlated with HOMA2-%B according to Spearman’s rank correlation analysis. Similarly, BW, BMI, AST levels, ALT levels, TG levels, HDL-C levels, FBG levels, serum C-peptide levels, renin activity, and the PAC were significantly correlated with HOMA2-IR. The multiple regression model revealed that BMI, HbA1c levels, and cortisol levels were significantly associated with HOMA2-%B. Furthermore, another multiple regression analysis revealed that ALT levels and the PAC were significant factors associated with HOMA2-IR in patients with untreated type 2 diabetes.

Our study demonstrated that the serum cortisol concentration was related to insulin secretion in patients with untreated type 2 diabetes. Cortisol is well known for stimulating hepatic gluconeogenesis [14]; however, it is also associated with insulin secretion. For example, higher serum cortisol levels are associated with decreased insulin secretion in the Japanese population [10], and higher morning cortisol levels are associated with increased FBG levels and reduced β-cell function in participants without type 2 diabetes [9]. Furthermore, therapeutic doses of prednisolone and hydrocortisone inhibit glucose-stimulated insulin secretion in human islets [15]. Several mechanisms have been proposed to explain how cortisol reduces insulin secretion. The overexpression of the glucocorticoid receptor in pancreatic β-cells suppresses insulin secretion [16, 17]. Furthermore, glucocorticoids inhibit insulin secretion by decreasing the effectiveness of cytoplasmic calcium ions in the secretory process of β-cells and by increasing islet glucose-6-phosphatase activity and glucose cycling [18, 19]. Therefore, cortisol may reduce insulin secretion by acting directly on β-cells.

Cortisol is widely known to impair insulin sensitivity at multiple sites, including the liver, skeletal muscle, and adipose tissue, leading to whole-body insulin resistance [20]. However, salivary cortisol levels are not associated with insulin resistance [21], and plasma cortisol levels do not differ significantly between controls and patients with obesity and insulin resistance [22]. In contrast, excess plasma glucocorticoids increase insulin resistance only in patients with obesity and insulin resistance but not in control participants [22]. In this study, the median (IQR) BMI and serum cortisol level were 25.4 (22.9–28.8) kg/m^2^ and 13.1 (9.5–16.6) µg/mL, respectively. Both values were within normal ranges. Therefore, serum cortisol levels might not be associated with insulin resistance in this study.

Although cortisol levels had a significantly negative correlation with HOMA2-%B in this study, reducing or blocking cortisol with pharmacotherapy to restore insulin secretion is an impractical treatment. However, psychological stress is a risk factor for type 2 diabetes [23], and the physiological stress response involves the activation of the hypothalamic‒pituitary‒adrenal axis and results in increased plasma cortisol levels [24]. If patients with type 2 diabetes who have higher cortisol levels experience psychological stress, care, such as mental health interventions, may reduce cortisol levels and restore insulin secretion. Therefore, measuring cortisol levels in patients with untreated type 2 diabetes has potential clinical utility.

Our study also demonstrated that the PAC is related to insulin resistance in patients with untreated type 2 diabetes. Some clinical studies have reported a relationship between high aldosterone levels and insulin resistance in patients without diabetes. Higher aldosterone levels exacerbate insulin resistance and is a risk factor for type 2 diabetes in participants without diabetes [11, 25]. In healthy young adults, high aldosterone levels (not ARR levels) are associated with insulin resistance, and aldosterone levels are higher in participants with metabolic syndrome than in those without it [26]. High aldosterone levels are also associated with increased visceral adipose tissue and liver fat accumulation [27]. Compared with essential hypertension, primary aldosteronism, a disease characterized by excessive plasma aldosterone concentrations, is associated with reduced insulin sensitivity and elevated β-cell function [28]. Furthermore, a decrease in aldosterone levels is associated with decreases in HOMA-IR and increases in adiponectin levels [29], and aldosterone blockade or treatment of primary aldosteronism alleviates insulin resistance [30–32]. These findings underscore the role of aldosterone in the development of insulin resistance.

Several mechanisms linking aldosterone to insulin resistance have been identified. Aldosterone decreases insulin-induced glucose uptake by decreasing the phosphorylation of mitogen-activated protein kinase and protein kinase b (Akt) and by promoting the degradation of insulin receptor substrate (IRS) 1 and IRS 2 in cultured adipocytes [33, 34]. In addition, mineralocorticoid receptor activation leads to proinflammatory action [35], and TNF-α inactivates insulin receptor substrate 1 (IRS-1) mediated by c-Jun N-terminal kinase [36]. Excess aldosterone impairs glucose uptake, oxidation, and insulin signal transduction by interfering with the expression of key proteins. Specifically, it causes defective expression of the insulin receptor (IR), IRS-1, Akt, Akt substrate of 160 kDa (AS160), and glucose transporter 4 (GLUT4) genes [37]. Furthermore, excess aldosterone reduces the phosphorylation of IRS-1, β-arrestin-2, and Akt in skeletal muscle [37]. Aldosterone also mediates the downregulation of phosphoenolpyruvate carboxykinase 1, perilipin, adiponectin, C1Q and the collagen domain in visceral adipose tissue, resulting in lower adiponectin levels and reduced insulin sensitivity in patients with primary aldosteronism [38, 39]. Thus, aldosterone is associated with insulin resistance in both adipocytes and skeletal muscle.

Aldosterone levels were significantly correlated with HOMA2-IR in this study. However, the r^2^ value in the multiple regression model was 0.0990, which is relatively low. Therefore, the predictive power of the model may not be strong. A clinical study revealed that spironolactone increases the levels of adiponectin, an insulin resistance marker, only in patients with type 2 diabetes whose HbA1c value is 8.0% or greater [40]. In this study, the median (IQR) HbA1c value was 10.3 (7.9–12.3)%, which means that the HbA1c value in many patients was 8.0% or greater. These results indicate that aldosterone may strongly influence insulin resistance in patients with type 2 diabetes whose HbA1c value is greater than 8.0%. Insulin resistance is related to not only aldosterone but also other factors, such as the amount of visceral adipose tissue. Therefore, we must be careful that the results of this study may not be applicable to all patients with untreated type 2 diabetes.

This study has several limitations. First, we could not analyze the duration of type 2 diabetes in this study. We could estimate the duration for some patients using data from health checks or blood test results from other hospitals or clinics; this information was not available for all participants. Therefore, we could not include the duration of type 2 diabetes in our analysis. Second, approximately 75% of the participants in this study were male. While no significant relationship was observed between sex and HOMA2-%B or HOMA2-IR, the study population was predominantly male. Finally, this was a single-center, retrospective study with a relatively small sample size. Future studies should include a larger number of patients with untreated type 2 diabetes and involve multiple medical centers across Japan and other countries. Despite these limitations, we demonstrated that the cortisol level is a predictor of HOMA2-%B and that the PAC is a predictor of HOMA2-IR in patients with untreated type 2 diabetes.

## Conclusion

Serum cortisol is associated with insulin secretion, and the plasma aldosterone concentration is associated with insulin resistance in patients with untreated type 2 diabetes. These results suggest that aldosterone blockade may be a therapeutic approach for alleviating insulin resistance in these patients. However, there are no reports that steroidal aldosterone blockers (spironolactone, eplerenone, and canrenone) ameliorate hyperglycemia in type 2 diabetes patients. Future studies using new nonsteroidal aldosterone blockades, such as esaxerenone and finerenone, should be conducted.

## Data Availability

No datasets were generated or analysed during the current study.

## Abbreviations

TNF: Tumor necrosis factor
HbA1c: Glycated hemoglobin
HOMA: Homeostatic model assessment
BW: Body weight
BMI: Body mass index
AST: Aspartate transaminase
ALT: Alanine transaminase
BUN: Blood urea nitrogen
eGFR: Estimated glomerular filtration rate
UA: Uric acid
TC: Total cholesterol
TG: Triglycerides
HDL-C: High-density lipoprotein-cholesterol
FBG: Fasting blood glucose
BP: Blood pressure
ACTH: Adrenocorticotropic hormone
PAC: Plasma aldosterone concentration
ECLIA: Electrochemiluminescence immunoassay
CLIA: Chemiluminescence immunoassay
EIA: Enzyme immunoassay
IQR: Interquartile range
VIF: Variance inflation factor
ARR: Aldosterone/renin activity ratio
FPG: Fasting plasma glucose
IRS: Insulin receptor substrate
IR: Insulin receptor
AS160: Akt phosphorylates its 160-kDa substrate
GLUT4: Glucose transporter 4
